# *Remarks on* Dr. Roberton's *Opinions Respecting Stricture in the Urethra, &c.*

**Published:** 1810-10

**Authors:** J. T. Williamson


### *Remarks on* Dr. Roberton's *Opinions respecting Stricture in the Urethra, &c.*

Dr. Roberton seems a man of strong natural powers of mind, both of these precious qualifications, are from other circumstances, quite lost in him; or rather, perverted and vitiated. A feeling mind cannot witness such a wreck, without sensations of pain and regret, for ability misapplied, is even worse than ignoble indolence, or complete idleness, the first being capable of leading myriads astray, whereas the latter can in general only be prejudicial in a great degree, to the individual himself.

We promised ourselves but little information, and less instruction a considerable time since, on seeing Dr. Roberton's principles announced, respecting stricture in the urethra; and the result of his inquiries, have not disappointed us. His mode of treating the subject, is so different from what we wished to hear, and what every rational being had a right to expect, that we are absolutely astonished, that he, or any other person, could have written so much as he has done, on the subject, in your Journal, and elsewhere, for several years past, without accidentally stumbling upon one valuable remark, or rational thought which might be applicable to any useful purpose; in short he must have shut his eyes, obscured judgment, and rendered his every sense torpid in the extreme, to the nature of his subject, merely for the purpose of bewildering his readers, and putting a mountain in labour, to bring forth a mouse; he must have robbed his reasoning faculties in an impregnable coat of mail, through which, from without, conviction, common sense, and acknowledged matter of fact, could never penetrate, while he himself possessed the unlimited power of deluging the world with his odd and unaccountable whims. He seems to have acted the part of the master of a puppet-shew, who sees all from within, outwards, while the gazers on, are only attracted, and put off their guard, by a little of his professional dexterity, which pleases the eye, but can never bring conviction to the mind.

We still hope, however, for his own sake, that Dr. Roberton's speculations respecting stricture, are, although they have seemingly taken some years to grow, only the first thoughts which he has bestowed on that universally prevailing, and most important complaint; and that he will soon become a convert to the only true and generally understood opinion entertained perhaps by every medical practitioner but himself, *that permanent stricture in the urethra, is, by general consent, and in general practice, known to be a disease of very common occurrence.* We have, in support of our opinions, common sense on our side, when we remark that it is more likely, in Dr. Roberton's speculations respecting stricture, that he is wrong, than that all the world except himself, should be burning strictures in the urethra, osophagus, &c. where they do not exist, and where one cause of disease, or of obstruction in these canals, is perpetually mistaken for another, by every one but himself. I say it is not likely, that in these points in particular, Dr. Roberton should be right, while we are all playing at blind man's buff, a groping in the dark, without knowing what we are about, unless we stumble on the right path by mere accident. Men capable of altering the established opinions of a whole world upon any very important point, but seldom in the present age appear among us. We admit, however, that when they do come in our way, they meet with great, and often unjust and merciless opposition, from the opposite party having acquired habits of thinking and acting, which they can only abandon with their own interest, and against their feelings of conviction. Thus they have usually much to strive with, before they ultimately succeed in establishing their views; but our opposition to Dr. Roberton's ideas of stricture, can scarcely be conceived to be of of this nature, or to arise from such a flattering idea of the business as this would be to Dr. Roberton. We wish to do him every justice, but the oddness of his notions, the gross absurdity and perverted stuff which we meet with in every paragraph of his writings, compel us rather to adhere to former, and, as *he* styles them, *absurd*, *barbarous*, and *cruel* doctrines and practices, than to acquiese in improbable, and I would almost say, impossible doctrines.

When such difficulty occurs to great and enlightened men, in the establishment of any important point, it is only because there is a seeming consistency in what they advance, but which yet cannot be properly comprehended and acted upon by the generality of people, at least for a length of time after the author has developed it. In other words, when a point is not ascertained to a demonstration, wherever different men are likely to view it in more ways than the right way, there opposition and clamour will be raised against it, which its intrinsic value alone can, and will ultimately silence. But a perusal of Dr. Roberton's notions, *on the diseases of the generative organs of both sexes*, will at once demonstrate that *they* possess no leading unextinguishable principle, which can, in spite of opposition, still blaze the brighter, and even at length make its most violent opponents become its warmest supporters. We shall, on the contrary, find that apparent plausability stands with him for matter of fact, and wrong principles and erronious doctrines are given us, instead of energetic, convincing, and self-evident demonstration, which at once strikes the mind. It is the blazing midnight faggot enticing us to it; but, in our passage, we unexpectedly find rocks and precipices to obstruct our progress; or it is the torch of Ariel leading us into Prospera's mire. It is the impracticable speculations of the dreamer, which only convince while we are deprived of the exercise of our faculties, but which dissolve into air when our reason returns, and when we consider the absurdity of supposing one individual right, and the whole world in error.

To conclude, Dr. Roberton's reasoning is so far from being like any thing we ever thought of, so different from that which we think likely to succeed, that we have no hesitation in pronouncing it the quintessence of extravagant folly, and the monster of an over-heated and hobbyhorsically vitiated imagination.

We cannot avoid expressing our wish, and we have little doubt that it will be the case, that the whole profession should concentrate their force in preventing an attempt at an overthrow of established opinions, and established doctrines—doctrines too, which we assert are successful in practice, by the extravagant, gant, absurd, and preposterous attempts of one who may employ his time more profitably for himself, gain and preserve more friends and supporters by his exertions, and be more successfully and beneficially employed in the alleviation of the distresses of human nature.

We hope these remarks will meet Dr. Roberton's eye before he advances farther, as author and instructor of the medical world. He informs us that he will soon publish a work on various diseases of the organs of generation, and we hope that on the principles of that particular part of it which we have now taken notice of, he will shape his doctrines more like acknowledged probability and the fashion of the day, and like the well-tried opinions of his professional contemporary brethren, than by at once, if I may be allowed the clumsy expression, attaching artificial wings to an ox, in expectation that that alone is necessary to make him fly.

*Oct.* 1809. J. T. WILLIAMSON.

